# Preoperative Progressive Pneumoperitoneum and Botulinum Toxin Type A in Patients With Large Parastomal Hernia

**DOI:** 10.3389/fsurg.2021.683612

**Published:** 2021-06-07

**Authors:** Fu-Xin Tang, Ning Ma, Xing-Xing Xie, Shuang Chen, Zhen Zong, Tai-Cheng Zhou

**Affiliations:** ^1^Department of Gastrointestinal Surgery and Hernia Center, The Sixth Affiliated Hospital of Sun Yat-sen University, Guangdong Institute of Gastroenterology, Guangdong Provincial Key Laboratory of Colorectal and Pelvic Floor Diseases, Supported by National Key Clinical Discipline, Guangzhou, China; ^2^Department of Gastrointestinal Surgery, The Second Affiliated Hospital of Nanchang University, Nanchang, China

**Keywords:** large parastomal hernia, botulinum toxin type A, preoperative progressive pneumoperitoneum, laparoscopic repair, loss of domain

## Abstract

**Background:** The combination of preoperative progressive pneumoperitoneum (PPP) and botulinum toxin type A (BTA) in adjuvant treatment of large parastomal hernia (LPH) has not been reported in the previous literature.

**Methods:** From February 2018 to June 2019, 16 patients were diagnosed with LPH in our hospital were included in this study. All patients received PPP and BTA treatment to expand abdominal volume and extend abdominal muscle before surgery. The laparoscopic Sugarbaker method was preferred for defect close.

**Results:** Before and after PPP and BTA, the mean volume of the parastomal hernia (VPH) was 1,522 and 1,644 cc, respectively (*P* < 0.01), and the mean volume of the abdominal cavity (VAC) was 5,847 and 9,408 cc, respectively (*P* < 0.01). The VPH/VAC ratio was decreased by an average of 8.4% after the combination management. And the lateral abdominal muscle length was increased by an average of 4.8 cm/side (*P* < 0.01). These patients underwent surgery successfully, and no hernia recurrence after (17.6 ± 2.4) months of follow-up.

**Conclusions:** The combination of PPP and BTA effectively expand the abdominal volume, decrease the risk of abdominal compartment syndrome (ACS) postoperatively, and beneficial to laparoscopic repair of LPH.

## Introduction

Despite recent advances in surgical techniques, repair of parastomal hernia (PSH) is still a huge challenge for surgeons following abdominal ostomy (1). The reported overall incidence rate of PSH varies widely in the literature effected by many factors, such as the type of stoma. Some literature has even reported the incidences as high as 32–44% (2, 3). However, permanent end colostomy has a higher incidence of PSH, estimated as up to 78% as revealed by computed tomography (CT) (4). These patients may present with leakage of fecal material from the appliance, resulting in contact dermatitis, and abdominal pain or incarceration or strangulation of the hernia, which may require emergency repair. These conditions seriously affect the patient's quality of life (5).

It is clear that surgery is the only form of treatment for PSH. The laparoscopic approach for PSH repair with mesh reinforcement has become a widely accepted procedure. The Sugarbaker technique has more lower recurrence than the keyhole (6). However, due to a history of complicated abdominal surgery or abdominal infection, these patients had severe abdominal adhesions, and parastatal hernia can easily lead to stoma obstruction, which obviously also increases the difficulty of the laparoscopic operation and the occurrence of complications. In particular, patients were diagnosed as large parastomal hernia (LPH), such as large incisional hernia (LIH) (loss of domain ratio >20%), are very likely to encounter the abdominal compartment syndrome (ACS) and other awful conditions postoperatively. Hence, preoperative management for LPH is crucial to avoid serious postoperative complications.

To avoid these accident, preoperative progressive pneumoperitoneum (PPP) was introduced by Moreno (7, 8). Preoperative progressive pneumoperitoneum can gradually expand the volume of the abdominal cavity (VAC) in the preoperative period and improve respiratory adaptation, so that LIH repair can be performed safely and the risk of ACS is greatly reduced (9). Recently, injection of botulinum toxin type A (BTA) to relax the abdominal wall muscles before LIH surgery became popular. Botulinum toxin type A is a neurotoxin isolated and purified from *Clostridium botulinum* type A bacteria that blocks the acetylcholine receptors, lead to reversible paralysis of the abdominal muscle that lasts for 6–9 months. Since 2009, successful use of BTA as a preparatory procedure for LIH, pain management after laparoscopic hernia repair and delayed abdominal wall reconstruction and so on have been reported (10–15).

The combination of PPP and BTA in adjuvant treatment of LPH has not been previously reported. Our aims is to explore the application value with the combination of PPP and BTA in adjuvant treatment of LPH, and hope to seek a more optimized way for the treatment of LPH.

## Methods

**Figure 1:**
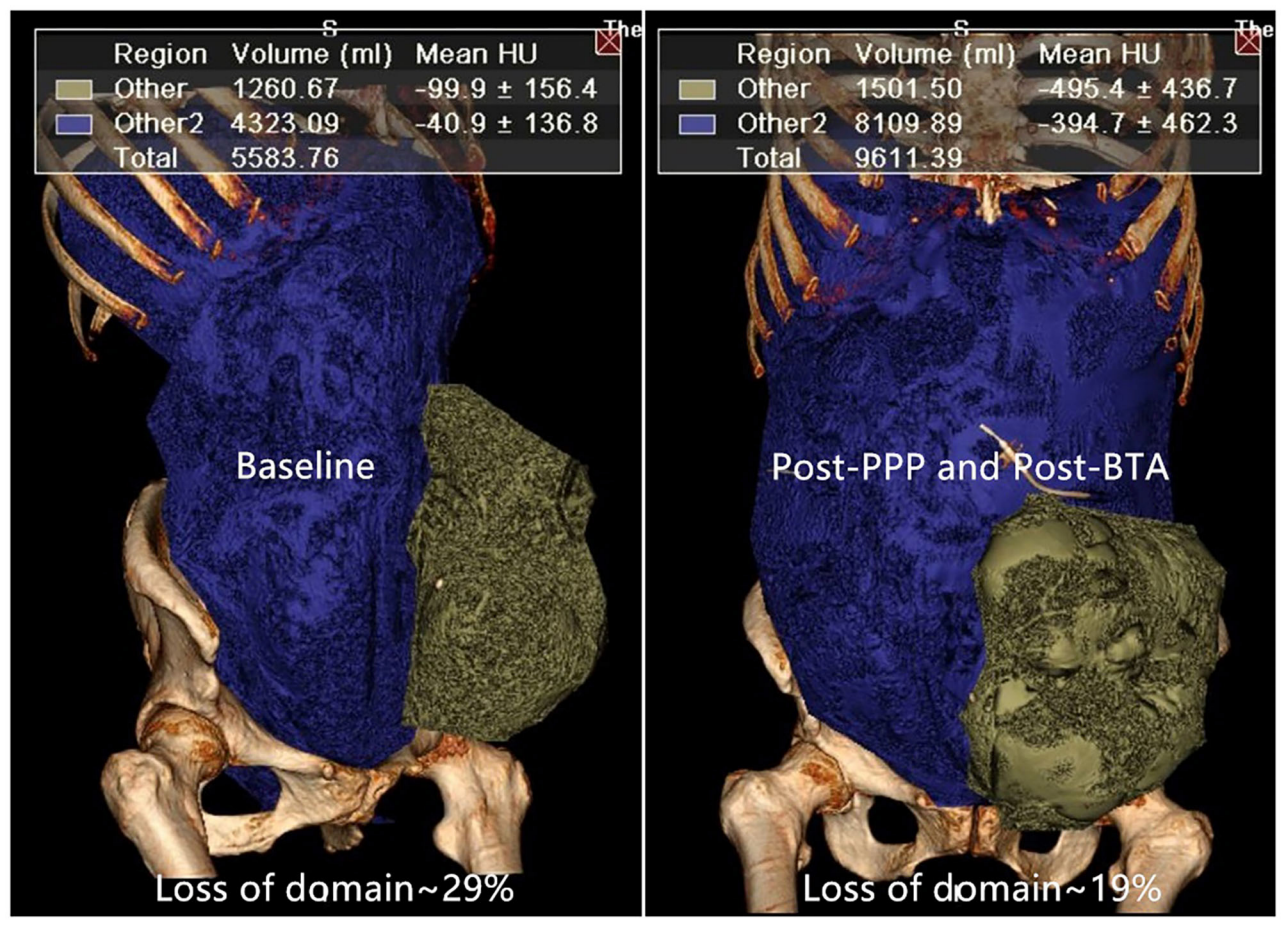
The abdominal volumes (VAC, VPH) were measured using specific software before and after preoperative techniques.

From February 2018 to June 2019, we retrospectively analyzed 16 patients with LPH (VPH/VAC ratio > 20%) by prospectively collected data. Changes in lateral abdominal muscle length and thickness, as well as volume of the parastomal hernia (VPH) and VAC, from before to after PPP and BTA were measured using computer tomography (CT) software (Figure 1). A radiologist specializing in the abdominal wall calculates the diameter and volume based on CT scans of the abdomen (16). Lateral muscle length was measured along the deep surface of the abdominal muscle complex from the lateral edge of the quadratus lumborum to the medial edge of rectus abdominis muscle on each side at the same spinal level. Further, muscle thickness was measured along the mid-axillary line from the deep surface of the transversus muscle to the superficial surface of the external oblique muscle. The patient was suspected of PSH and was requested preoperatively for a CT scan. As long as the VPH/VAC ratio was ≥20%, the patient would be enrolled in this study and specific management was performed simultaneously 2–3 weeks prior to surgery. Parastomal hernia was classified according to the European Hernia Society (EHS) classification (17).

In order to promote postoperative recovery, smokers are advised to stop smoking immediately until recovery from surgery; for obese patients, diet and exercise are recommended to reduce obesity.

### BTA Protocol

Botulinum Toxin Type A injections were performed simultaneously on the admitted patients. All patients received a total dose of 150 units of BTA (Botulinum Toxin Type A for injection, BOTOX^®^, Allergan, Ireland), which was diluted to 2 units/cc with 0.9% saline and the total was divided into six equal parts. All patients received injections bilaterally. With the patient lying on one side, using real-time ultrasound guidance, three sites were identified on each side of the abdomen as previously identified (14). To compensate for severe retraction of the abdominal muscles or the lack of a muscular layer, some injection sites were variable. Then, 12 cc of the diluted BTA was injected at the six points of the three lateral oblique muscles (transversus abdominis, internal oblique, and external oblique), and the patient was returned to a supine position.

### PPP Protocol

An PPP catheter was placed preferably in the upper left quadrant under ultrasound guidance and distant from the hernia sac and previous incision sites in the admitted patient. We initially insufflated 200 cc of air, a standing X-ray was subsequently taken to determine the catheter was in the abdominal cavity. This pneumoperitoneum procedure lasts about 2 weeks for gradual expand capacity. Subsequently, a total of 400 cc daily was insufflated, divided into 200 cc in the morning and 200 cc in the afternoon, using a 50-cc syringe. The patients were discharged after 24–48 h to continue PPP at home under outpatient management. During the period of PPP administration, the patients wore an abdominal support belt to compress the hernia sac and were encouraged to improve their respiratory function. After insufflation, the lateral abdominal muscle length, thickness, and abdominal volume were measured again. We monitored the patient's insufflation every day and asked the patients if they had any symptoms such as abdominal pain, dyspnea, or shoulder pain.

### Surgical Procedure

All patients underwent PSH repair via a laparoscopic approach using the Sugarbaker technique (18, 19). The operation was carried out under general anesthesia, and an indwelling urinary catheter was routinely inserted after intubation. Initial insufflation was performed with the use of an intraperitoneal catheter. Usually, two 5-mm working ports and one 12-mm viewing port were inserted on one side of the abdomen. Adhesiolysis was required to fully discover the hernia defect, and the hernia contents were pulled back into the abdominal cavity. Using single-strand non-absorbable sutures (2-0 Prolene^®^) to close defect, and the gap for intestinal stoma was reserved and the hernia defect was reinforced with a composite polyester mesh (PCOPM 20, Sofradim, France). The mesh placement was intraperitoneal Sugarbaker mesh, and was fixed with non-absorbable tacks (Covidien 11, USA). In the end, the trocar hole was closed and no drainage was placed.

Postoperatively, the patients wore abdominal bandages for at least 6 months. Some patients developed severe complications require ventilatory support were transferred to the intensive care unit for further treatment. Abdominal pressures were monitored directly by monitoring bladder pressure for 3 days or longer if indicated. Intravenous antibiotics were continued for 72 h or longer if indicated, and enoxaparin was routinely given for deep vein thromboembolic prophylaxis continued throughout admission.

### Statistical Analysis

Descriptive statistics, including means and standard deviations, were used for continuous variables. Changes in lateral abdominal muscle length as well as VPH and VAC before and after preoperative techniques were measured. Univariate analysis was carried out using Student's *t*-test to analyze quantitative variables, and the chi-square or Fisher's test was used if the data were dichotomous. All statistics were carried out with the aid of SPSS version 20.0 software. A *P*-value <0.05 was considered to be a statistical difference.

## Results

Between February 2018 and June 2019, 16 patients (9 males and 7 females) underwent our protocol using both PPP and BTA injections. The details of the 16 patients are listed in Table 1. The mean age was 63.4 ± 9.6 years (range, 45–77 years), and the mean body mass index was 25.7 ± 3.0 kg/m^2^ (range, 22.4–34.7 kg/m^2^). The mean time since occurrence of PSH was (28.6±18.9) months (range, 9–78 months). Three of the sixteen patients had one previous failed repair and two patients had two or more failed repairs. According to PSH grade, two patients were classified as Grade I (12.5%), eleven as Grade III (68.8%), and three as Grade IV (18.8%).

**Table 1:** Demographics and characteristics of the patients with large parastomal hernias.

**Characteristics**	**PPP and BTA (*n* = 16)**
Mean age	63.4 ± 9.6 (45–77)
Sex	
Male	9 (56.3)
Female	7 (43.8)
Median previous mesh-repair	0.6 ± 1.0 (0–4)
Stoma type	
Colostomy	9 (56.3)
Ileostomy	6 (37.5)
Ileal orthotopic neobladder	1 (6.3)
First abdominal surgery	
Rectectomy (Mile's surgery)	9 (56.3)
Colectomy	6 (31.3)
Radical cystectomy	1 (6.3)
Evolution time of hernia, months	28.6 ± 18.9 (9–78)
Stoma disease	
Enterostomy prolapse	0 (0)
Incomplete intestinal obstruction	7 (44.8)
PSH grade	
Grade I	2 (12.5)
Grade III	11 (68.8)
Grade IV	3 (18.8)
Obesity (BMI > 30)	1 (6.3)
History of active smoking	4 (25)
Other comorbidities	
Diabetes	3 (19.0)
Hypertension	5 (31.0)
COPD	2 (13.0)
History of abdominal sepsis	6 (38.0)

Four patients (25%) were smokers, two patients (13%) had chronic obstructive pulmonary disease, three patients (19%) had diabetes, and five patients (31%) had hypertension. Six patients (38%) had a history of abdominal sepsis, and seven patients (44%) had preoperative incomplete intestinal obstruction.

**Figure 2:**
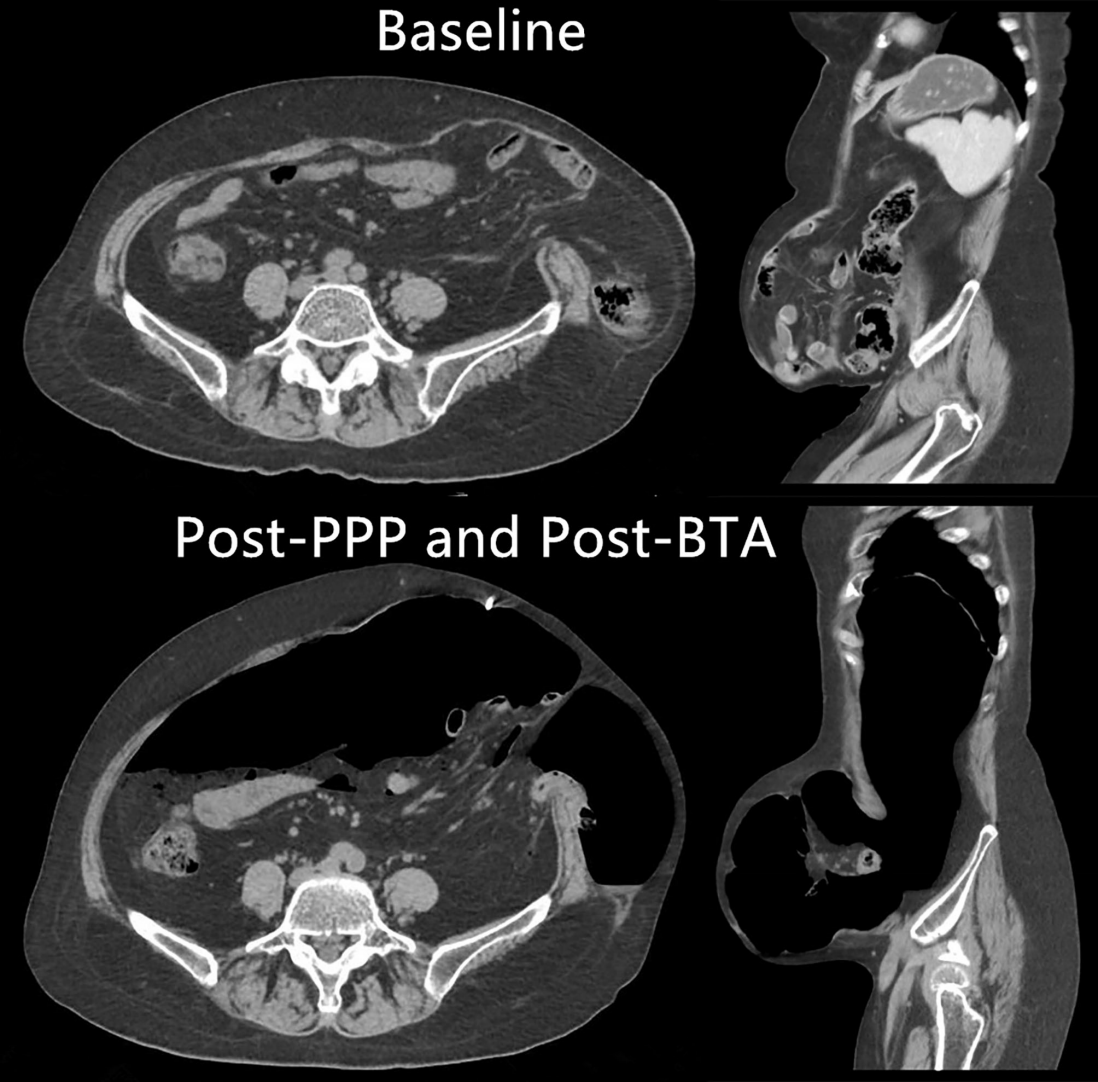
Abdominal CT picture of before and after PPP and BTA treatments demonstrating spontaneously reinstate the herniated contents.

**Figure 3:**
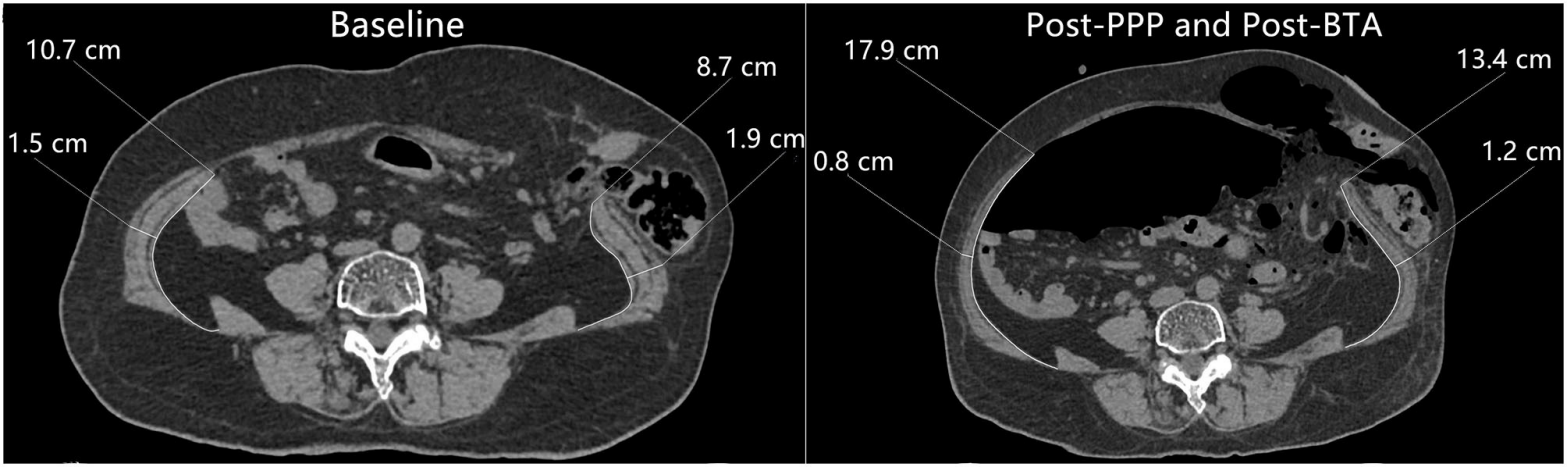
Comparison of preoperative axial computed tomography picture before and after preoperative techniques. There is significant elongation and thinning of the lateral abdominal muscles.

The mean insufflated amount of air was 6,325 ± 748 cc (range, 5,400–7,800 cc) over a period of 15.9 ± 1. 7 days (range, 14–19 days). The mean time of BTA administration was 16.3 ± 1.9 days (range, 14–21 days). Before and after PPP, respectively, the mean VPH was 1,522 ± 377 cc (range, 963–2,567 cc) and 1,644 ± 372 cc (range, 1,124–2,576 cc) (*P* < 0.01), the mean VAC was 5,847 ± 1,107 cc (range, 4,323–8,834 cc) and 9,408 ± 1,178 cc (range, 7,356–11,648 cc) (*P* < 0.01), and the VPH/VAC ratio (LODH ratio) was 26.1 ± 4.5% (range, 20.2–38.9%) and 17.7 ± 4.8% (range, 13.8–34.4%) (Table 2). The VPH/VAC ratio was decreased by 8.4% after the combination management, a statistically significant reduction was obtained (Figure 1). Abdominal CT pictures of before and after PPP showed spontaneous reinstatement of the herniated contents (Figures 2, 3).

**Table 2:** Comparison of peritoneal volumes.

	**Before PPP+BTA**	**After PPP +BTA**	***P*-value**
	**(range)**	**(range)**	
VPH (cc)	1,683 ± 465	1,817 ± 458	<0.01
	(876–2,536)	(986–2,768)	
VAC (cc)	6,961 ± 2,208	10,471 ± 2,122	<0.01
	(4,000–12,047)	(6,672–13,909)	
VPH/VAC ratio	24.8 ± 5.7	17.3 ± 2.7	<0.01
	(20.5–50.9)	(13.4–26.9)	

*P-value derived from Student's t-test. PPP, progressive preoperative pneumoperitoneum; BTA, botulinum toxin A; VPH, parastomal hernia volume; VAC, abdominal cavity volume*.

A comparison of CT images before and after administration of the combination approach showed increased lateral abdominal wall muscle length and simultaneous decreased muscle thickness (Figure 3). There was a statistically significant increase in abdominal muscle length from 13.1 ± 2.8 cm/side (range, 9.3–20.8 cm/side) before application of the preoperative techniques to 17.9 ± 2.8 cm/side (range, 13.8–25.9 cm/side) after the combination approach (*P* < 0.01), indicating the lateral abdominal muscle length was increased by an average of 4.8 cm/side (range, 3.4–6.4 cm/side) (Table 3). Meanwhile, lateral abdominal muscle thickness was reduced by an average of 6.7 mm (range, 3.5–13.5 mm).

**Table 3:** Results of all 16 patients undergoing PPP treatment and pre-operative BTA injection.

**Patient**	**Hernia size (cm)**	**Length (left) (cm)**			**Length (right) (cm)**		
		**Pre-PPP/BTA**	**Post-PPP/BTA**	**Difference**	**Pre-PPP/BTA**	**Post-PPP/BTA**	**Difference**
PT-1	6 × 8	8.7	13.4	5.2	10.7	17.9	5.9
PT-2	4 × 5	12.3	15.6	3.3	13.0	17.5	4.5
PT-3	5 × 7	12.9	16.0	4.9	16.4	20.6	4.2
PT-4	7 × 11	14.6	21.1	6.5	12.6	15.8	3.2
PT-5	8 × 8	19.2	25.6	6.4	22.4	26.2	3.8
PT-6	9 × 10	9.4	13.4	4.0	9.1	14.2	5.1
PT-7	8 × 8	10.9	15.2	4.3	13.6	18.4	4.8
PT-8	11 × 9	12.6	18.1	5.5	13.5	19.5	6.0
PT-9	10 × 10	14.7	17.8	3.1	16.6	20.2	3.6
PT-10	10 × 9	8.7	15.9	7.2	16.5	22.1	5.6
PT-11	8 × 9	16.1	23.0	6.9	15.0	20.2	4.8
PT-12	7 × 9	10.1	15.5	4.4	11.4	16.6	5.2
PT-13	6 × 9	11.8	15.1	3.3	11.1	16.8	5.7
PT-14	7 × 10	12.3	16.7	4.4	13.1	18.5	5.4
PT-15	10 × 11	14.9	18.8	3.9	11.9	15.0	3.1
PT-16	9 × 10	11.1	15.6	4.5	10.6	16.1	5.5
Mean				4.8			4.9

*Comparison of lateral abdominal muscle length before and after the preoperative combined use of BTA injection and PPP treatment are show, as measured on CT imaging. All patients received bilateral BTA injections. PT, patient*.

Complications associated with PPP occurred in 13.3% of patients. No complications occurred during the administration of BTA. One patient (6.7%) developed subcutaneous emphysema, which disappeared spontaneously. One patient had shoulder pain (6.7%), which was tolerable. Pneumothorax, bowel injury and so on did not occur. And these complications do not require special treatment.

For LPH repair, all patients underwent the laparoscopic approach with the Sugarbaker technique. The mean operation time was 183 ± 25 min (range, 130–230 min). During abdominal adhesiolysis, we performed one bowel resection because of accidental intestinal perforation, but no other accidents occurred postoperatively. Postoperatively, the mean bladder pressure was 12.8 ± 1.8 cm H_2_O (range, 7–15 cm H_2_O) (1 cm H_2_O = 0.098 kPa). The postoperative morbidity rate related to the operation was 30.4%. One patient developed intraperitoneal bleeding, which improved after embolization. One patient had a cerebral infarction and was transferred to the Department of Neurology for treatment. Two patients developed delayed defecation or incomplete bowel obstruction. One patient developed pneumonia and was treated with antibiotics. No patient had ACS or cardiopulmonary failure. Average postoperative hospital stay was 9.9 ± 5.0 days (range, 6–26 days). The mean follow-up time was 17.6 ± 2.4 months (range, 13–21 months), with no early recurrences.

## Discussion

Parastomal hernia is a common accident after ostomy and is related to considerable morbidity and impact on quality of life, especially in patients with a permanent ostomy (20). The recurrence rate of PSH is about 70% after simple fascial close and 20% after parastomal mesh repair (21–23). A meta-analysis suggested that the laparoscopic Sugarbaker method was related to lower recurrence rate of 10.0% compared with the keyhole mesh with a recurrence rate of up to 28% (24). A multicenter cohort study including 61 patients treated by the laparoscopic Sugarbaker technique also reported a low recurrence rate of 6.6% with a 26-month follow-up (25). Therefore, we use this technique for all patients in our center. There is no definition of LPH in the literature, and therefore we defined LPH as a LIH with LODH ratio of ≥20% (VPH/VAC ≥ 20%). During surgical repair of the unprepared abdomen with a large ventral hernia, forced reduction of the hernia contents can result in awful pathophysiological complications (26). Hence, for a patient with LPH with LODH prior to repair, adequate preparation is crucial. In recent years, the combined use of PPP and BTA is recommended to avoid these risks in LODH repair (27).

Preoperative progressive pneumoperitoneum is proposed for use in patients with large hernias (28), or forced reduction of the hernia contents can lead to the patient encounter with ACS (29). Preoperative progressive pneumoperitoneum can increase the abdominal volume, facilitate reintroduction of the hernia sac, and reduce ACS and the involvement of respiratory function, which enables the LODH repair to be safely performed (9, 30). This technique is being used more widely in abdominal wall hernia repair, and many related studies have reported good results (9, 30–34). However, there is no agreement on the total amount of air insufflation and maintenance time for PPP techniques (12). In our experience, we insufflate four times the volume of the hernia sac. This volume is introduced over approximately 2 weeks for progressive adaptation. In a study of 45 patients, Renard et al. reported a significant increase in abdominal cavity volume of 53% (*P* < 0.0001) and a minimum of 15 days of PPP (34). Dumont et al. reported an increase in abdominal muscle length in 18 LIH patients with a mean amount of 12.8 L of air insufflated with a mean period of 14.8 days, but PPP had an equivalent effect on the width of the hernial orifice (9).

This technique has many other advantages. Preoperative progressive pneumoperitoneum can perform pneumatic adhesiolysis prior to repair, which decreases the amount of dissection during the operation (7, 26, 31). Preoperative progressive pneumoperitoneum also benefits diaphragmatic function (28), and allows the spontaneous reduction of the hernia contents, with the remaining adhesions clearly discernible (26) (Figures 2, 3). Preoperative CT after PPP enables the surgeon to locate areas on the abdominal wall that are free of adhesions, and thus initial entry of the trocar into the abdomen is safe, because blind entry can lead to inadvertent enterotomy (26). This procedure can be performed on outpatients, which reduces the burden on patients and saves medical resources. Our study also demonstrates the safety of the procedure.

Ibarra et al. first reported the application of BTA in 12 patients with abdominal hernias, 10 patients with an mean reduction of (5.25 ± 2.32) cm in transverse hernia defect were obtained (*P* < 0.001) after 4 weeks of BTA injection, and hernia repair was performed with no recurrences (10). Botulinum toxin type A causes temporary flaccid paralysis, leading to elongation and thinning of the retracted muscles of the abdomen. The maximum effect is achieved in 2 weeks, and lasts for 6–9 months (35). There are some studies about preoperatively BTA injection to assist incisional hernia repair, and was proved that preoperative BTA injection is a safe and effective technique (36–38). Botulinum toxin type A injection is also used for pain management after incisional hernia repair (14).

The first study reporting the preoperative combination of PPP and BTA in 45 patients with LIH was published by Bueno-Lledó et al. (12). And the VIH/VAC ratio was reduced by an average of 14% after the combination management (*P* < 0.05), and hernia defect closure was achieved in all patients, with only two cases of hernia recurrence. In 2018, Bueno-Lledó et al. reported the preoperative combination of PPP and BTA in 70 patients with LODH, and good results were obtained. Transverse hernia defect in the two studies decreased by an average of 0.8 and 0.9 cm, respectively, but the changes were not statistically significant.

Our group considers that BTA may be useful to elongate retracted muscles without altering their anatomical constitution, permitting primary fascial closure without tension (10, 11). The main function of PPP is to significantly expand abdominal volume, thus reducing LODH. Thus, the characteristics of BTA may perfectly complement the function of PPP. Considering the advantages of PPP combined with BTA, we attempted to apply this approach to the preoperative preparation of LPH repair. In this study, VPH and VAC with an average increase of 123 cc and 3,561 cc after PPP (*p* < 0.01), respectively, which represented a mean increase of 64% and was superior to mean VPH before PPP (1,522 cc). Furthermore, the VPH/VAC ratio was reduced from 26.1 to 17.7% after PPP (*p* < 0.01). This reduction is significantly important, because it is conducive to the reintroduction of the hernia contents, decreasing the postoperative incidence of ACS. The lateral abdominal muscles length was increased by an average of 4.8 cm/side, and this increase can eliminate the need for the component separation technique. In addition, by using this method, the muscle structure has not been destroyed, which is important for subsequent surgical treatment (37).

## Limitations and Strengths

For the first time, we reported the preoperative combined use of PPP and BTA to assist laparoscopic treatment of LPH, and achieved a relatively satisfactory effect, which provides a new way for clinical treatment of LPH. However, the main shortcoming of the study was the small number of cases included, the efficacy of PPP or BTA treatment alone was not compared with the combination of PPP and BTA and the follow-up time was short.

## Conclusions

Preoperative progressive pneumoperitoneum and Botulinum toxin type A are useful techniques in the repair of LPH. They passively expand the abdominal capacity, allowing the viscera to re-establish the right of domain. The effect of BTA lasts for 6–9 months after surgery, ensuring the complete healing of the incision and greatly reducing the chance of recurrence. Most importantly, the combined use of PPP and BTA decreases the risk of postoperative ACS and enables laparoscopic repair of LPH.

## Data Availability Statement

The raw data supporting the conclusions of this article will be made available by the authors, without undue reservation.

## Ethics Statement

The studies involving human participants were reviewed and approved by Ethics Committee of the Sixth Affiliated Hospital of Sun Yat-sen University. The patients/participants provided their written informed consent to participate in this study.

## Author Contributions

We acknowledge the kind assistance of ZZ in designing the study and NM and X-XX in editor the manuscript. Furthermore, we would like to acknowledge T-CZ and SC for their language editing and designing this study. All authors listed have made a substantial, direct and intellectual contribution to the work, and approved it for publication.

## Conflict of Interest

The authors declare that the research was conducted in the absence of any commercial or financial relationships that could be construed as a potential conflict of interest.

## References

[1] Lopez-Borao J Madrazo-Gonzalez Z Kreisler E Biondo S. Prevention of parastomal hernia after abdominoperineal excision with a prophylactic three-dimensional funnel mesh. Colorectal Dis. (2019) 21:1326–34. 10.1111/codi.14738 31230409

[2] Vierimaa M Klintrup K Biancari F Victorzon M Carpelan-Holmström M Kössi J. Prospective, randomized study on the use of a prosthetic mesh for prevention of parastomal hernia of permanent colostomy. Dis Colon Rectum. (2015) 58:943–9. 10.1097/DCR.0000000000000443 26347966

[3] Janes A Cengiz Y Israelsson LA. Preventing parastomal hernia with a prosthetic mesh: a 5-year follow-up of a randomized study. World J Surg. (2009) 33:118–21; discussion 122–3. 10.1007/s00268-008-9785-4 19011935

[4] Carne PW Robertson GM Frizelle FA. Parastomal hernia. Br J Surg. (2003) 90:784–93. 10.1002/bjs.4220 12854101

[5] Kald A Juul KN Hjortsvang H Sjodahl RI. Quality of life is impaired in patients with peristomal bulging of a sigmoid colostomy. Scand J Gastroenterol. (2008) 43:627–33. 10.1080/00365520701858470 18415759

[6] Hansson BM Bleichrodt RP de Hingh IH. Laparoscopic parastomal hernia repair using a keyhole technique results in a high recurrence rate. Surg Endosc. (2009) 23:1456–9. 10.1007/s00464-008-0253-x 19118435

[7] Moreno IG. Chronic eventrations and large hernias; preoperative treatment by progressive pneumoperitomeum; original procedure. Surgery. (1947) 22:945–53. 20271801

[8] Goni Moreno I. [Pneumoperitoneum applied to the surgical preparation of large chronic eventrations]. Prensa Med Argent. (1971) 58:1037–41. 5096685

[9] Dumont F Fuks D Verhaeghe P Brehant O Sabbagh C Riboulot M. Progressive pneumoperitoneum increases the length of abdominal muscles. Hernia. (2009) 13:183–7. 10.1007/s10029-008-0436-3 18949443

[10] Ibarra-Hurtado TR Nuno-Guzman CM Echeagaray-Herrera JE Robles-Velez E de Jesus Gonzalez-Jaime J. Use of botulinum toxin type a before abdominal wall hernia reconstruction. World J Surg. (2009) 33:2553–6. 10.1007/s00268-009-0203-3 19771472

[11] Zielinski MD Goussous N Schiller HJ Jenkins D. Chemical components separation with botulinum toxin A: a novel technique to improve primary fascial closure rates of the open abdomen. Hernia. (2013) 17:101–7. 10.1007/s10029-012-0995-1 23001400

[12] Bueno-Lledó J Torregrosa A Ballester N Carreño O Carbonell F Pastor PG. Preoperative progressive pneumoperitoneum and botulinum toxin type A in patients with large incisional hernia. Hernia. (2017) 21:233–43. 10.1007/s10029-017-1582-2 28124308

[13] Elstner KE Jacombs AS Read JW Rodriguez O Edye M Cosman PH. Laparoscopic repair of complex ventral hernia facilitated by pre-operative chemical component relaxation using botulinum toxin A. Hernia. (2016) 20:209–19. 10.1007/s10029-016-1478-6 26951247

[14] Smoot D Zielinski M Jenkins D Schiller H. Botox A injection for pain after laparoscopic ventral hernia: a case report. Pain Med. (2011) 12:1121–3. 10.1111/j.1526-4637.2011.01147.x 21668748

[15] Bueno-Lledo J Torregrosa A Jimenez R Pastor PG. Preoperative combination of progressive pneumoperitoneum and botulinum toxin type A in patients with loss of domain hernia. Surg Endosc. (2018) 32:3599–608. 10.1007/s00464-018-6089-0 29450631

[16] Tanaka EY Yoo JH Rodrigues AJ Jr Utiyama EM Birolini D Rasslan S. A computerized tomography scan method for calculating the hernia sac and abdominal cavity volume in complex large incisional hernia with loss of domain. Hernia. (2010) 14:63–9. 10.1007/s10029-009-0560-8 19756913

[17] Smietanski M Szczepkowski M Alexandre JA Berger D Bury K Conze J. European Hernia Society classification of parastomal hernias. Hernia. (2014) 18:1–6. 10.1007/s10029-013-1162-z 24081460 PMC3902080

[18] Sugarbaker PH. Prosthetic mesh repair of large hernias at the site of colonic stomas. Surg Gynecol Obstet. (1980) 150:576–8. 7361254

[19] Mancini GJ McClusky DA III Khaitan L Goldenberg EA Heniford BT Novitsky YW. Laparoscopic parastomal hernia repair using a nonslit mesh technique. Surg Endosc. (2007) 21:1487–91. 10.1007/s00464-007-9419-1 17593454

[20] Antoniou SA Agresta F Garcia Alamino JM Berger D Berrevoet F Brandsma HT. European Hernia Society guidelines on prevention and treatment of parastomal hernias. Hernia. (2018) 22:183–98. 10.1007/s10029-017-1697-5 29134456

[21] Brandsma HT Hansson BM Aufenacker TJ van Geldere D Lammeren FM Mahabier C. Prophylactic mesh placement during formation of an end-colostomy reduces the rate of parastomal hernia: short-term results of the Dutch PREVENT-trial. Ann Surg. (2017) 265:663–9. 10.1097/SLA.0000000000001903 27471840

[22] De Raet J Delvaux G Haentjens P Van Nieuwenhove Y. Waist circumference is an independent risk factor for the development of parastomal hernia after permanent colostomy. Dis Colon Rectum. (2008) 51:1806–9. 10.1007/s10350-008-9366-5 18483825

[23] Hansson BM Slater NJ van der Velden AS Groenewoud HM Buyne OR de Hingh IH. Surgical techniques for parastomal hernia repair: a systematic review of the literature. Ann Surg. (2012) 255:685–95. 10.1097/SLA.0b013e31824b44b1 22418006

[24] DeAsis FJ Lapin B Gitelis ME Ujiki MB. Current state of laparoscopic parastomal hernia repair: a meta-analysis. World J Gastroenterol. (2015) 21:8670–7. 10.3748/wjg.v21.i28.8670 26229409 PMC4515848

[25] Hansson BM Morales-Conde S Mussack T Valdes J Muysoms FE Bleichrodt RP. The laparoscopic modified Sugarbaker technique is safe and has a low recurrence rate: a multicenter cohort study. Surg Endosc. (2013) 27:494–500. 10.1007/s00464-012-2464-4 23052490 PMC3580038

[26] Elstner KE Read JW Rodriguez-Acevedo O Ho-Shon K Magnussen J Ibrahim N. Preoperative progressive pneumoperitoneum complementing chemical component relaxation in complex ventral hernia repair. Surg. Endosc. (2017) 31:1914–22. 10.1007/s00464-016-5194-1 27572061

[27] Alam NN Narang SK Pathak S Daniels IR Smart NJ. Methods of abdominal wall expansion for repair of incisional herniae: a systematic review. Hernia. (2016) 20:191–9. 10.1007/s10029-016-1463-0 26860729

[28] Willis S Schumpelick V. Use of progressive pneumoperitoneum in the repair of giant hernias. Hernia. (2000) 4:105–11. 10.1007/BF02353758

[29] Munegato G Grigoletto R Brandolese R. Respiratory mechanics in abdominal compartment syndrome and large incisional hernias of the abdominal wall. Hernia. (2000) 4:282–5. 10.1007/BF01201084

[30] Sabbagh C Dumont F Fuks D Yzet T Verhaeghe P Regimbeau JM. Progressive preoperative pneumoperitoneum preparation (the Goni Moreno protocol) prior to large incisional hernia surgery: volumetric, respiratory and clinical impacts. A prospective study. Hernia. (2012) 16:33–40. 10.1007/s10029-011-0849-2 21773758

[31] Mayagoitia JC Suarez D Arenas JC Diaz de Leon V. Preoperative progressive pneumoperitoneum in patients with abdominal-wall hernias. Hernia. (2006) 10:213–7. 10.1007/s10029-005-0040-8 16261394

[32] McAdory RS Cobb WS Carbonell AM. Progressive preoperative pneumoperitoneum for hernias with loss of domain. Am Surg. (2009) 75:504–8; discussion 508–9. 10.1177/000313480907500609 19545099

[33] Lopez Sanclemente MC Robres J Lopez Cano M Barri J Lozoya R López S. [Progressive preoperative pneumoperitoneum in patients with giant hernias of the abdominal wall]. Cir Esp. (2013) 91:444–9. 10.1016/j.cireng.2012.08.001 23473433

[34] Renard Y. Lardiere-Deguelte S de Mestier L Appere F Colosio A Kianmanesh R. Management of large incisional hernias with loss of domain: a prospective series of patients prepared by progressive preoperative pneumoperitoneum. Surgery. (2016) 160:426–35. 10.1016/j.surg.2016.03.033 27262533

[35] Dressler D. Clinical applications of botulinum toxin. Curr Opin Microbiol. (2012) 15:325–36. 10.1016/j.mib.2012.05.012 22770659

[36] Zendejas B Khasawneh MA Srvantstyan B Jenkins DH Schiller HJ Zielinski MD. Outcomes of chemical component paralysis using botulinum toxin for incisional hernia repairs. World J Surg. (2013) 37:2830–7. 10.1007/s00268-013-2211-6 24081529

[37] Farooque F Jacombs AS Roussos E Read JW Dardano AN Edye M. Preoperative abdominal muscle elongation with botulinum toxin A for complex incisional ventral hernia repair. ANZ J Surg. (2016) 86:79–83. 10.1111/ans.13258 26245344

[38] Elstner KE Read JW Rodriguez-Acevedo O Cosman PH Dardano AN Jacombs AS. Preoperative chemical component relaxation using Botulinum toxin A: enabling laparoscopic repair of complex ventral hernia. Surg Endosc. (2017) 31:761–8. 10.1007/s00464-016-5030-7 27351658

